# Executive and motivational pathways to ADHD traits in the general population: a structural equation model of working memory, attention, delay discounting, and decision-making

**DOI:** 10.1186/s40359-026-04279-x

**Published:** 2026-03-06

**Authors:** Masanaga Ikegami, Michiko Sorama, Aya Katayama, Hidetoshi Omiya

**Affiliations:** 1 Department of Psychology, Asahikawa Medical University, 2-1-1-1 Midorigaoka-Higashi, Asahikawa, Hokkaido, 078-8510 Japan; 2 Department of Psychology, Kyoto Notre Dame University, Kyoto, Japan; 3 Department of Cognitive Neuroscience, Cognitive Mechanisms Laboratories, Advanced Telecommunications Research Institute International (ATR), Kyoto, Japan; 4 Faculty of Psychology, Sapporo Gakuin University, Sapporo, Japan

**Keywords:** ADHD traits, Executive functions, Delay discounting, Decision-making, Structural equation modeling

## Abstract

**Background:**

Research has associated attention-deficit/hyperactivity disorder (ADHD) with difficulties in executive functions, such as working memory, sustained attention, and inhibitory control, as well as with motivational alterations, including steep delay discounting. However, few studies have examined how these processes jointly contribute to ADHD-related decision-making within an integrative framework. To address this gap, the present study examined delay discounting and executive functions as predictors of decision-making performance under risk, with the aim of clarifying how suboptimal decision-making may be expressed in individuals with elevated ADHD traits.

**Methods:**

Sixty-three university students completed a behavioral battery assessing working memory, sustained attention, response inhibition, and decision-making on the Cambridge Gambling Task (CGT). Delay discounting was measured using the five-trial adjusting delay task, and ADHD traits were assessed with the Adult ADHD Self-Report Scale. Hypothesized relationships among these variables were examined using structural equation modeling (SEM).

**Results:**

ADHD trait scores were negatively correlated with the CGT indices of decision-making quality and risk adjustment. Working memory errors were most strongly associated with poorer CGT performance, with sustained attention and response inhibition showing weaker associations. SEM indicated sequential indirect pathways linking steeper delay discounting to higher ADHD traits through reduced sustained attention, increased working-memory errors, and poorer CGT performance.

**Conclusions:**

The findings support a multi-pathway account of ADHD traits, suggesting that motivational (delay discounting) and executive (working memory, attention, inhibition) mechanisms jointly contribute to suboptimal decision-making under risk. This integrative framework advances understanding of the cognitive and motivational mechanisms underlying ADHD-related decision processes and may inform future assessment and intervention approaches.

**Supplementary Information:**

The online version contains supplementary material available at 10.1186/s40359-026-04279-x.

## Background

Attention-deficit/hyperactivity disorder (ADHD) is one of the most common neurodevelopmental conditions, affecting about 3–7% of children and 2–3% of adults worldwide [1–3]. Although the core symptoms of inattention and hyperactivity typically emerge in childhood, many individuals continue to exhibit residual difficulties in attention, motivational and emotional regulation, and executive functioning well into adulthood [4, 5]. ADHD is now widely regarded not as a discrete categorical disorder but as reflecting dimensional traits of attention and behavioral regulation that vary continuously across the general population [4]. Although only a minority of adults meet the full diagnostic criteria, estimates suggest that up to 7% of adults show significant ADHD symptoms [3], supporting this dimensional perspective. Subclinical ADHD traits, observed in about 10% of adults [6], have been linked to psychosocial and cognitive difficulties [7, 8]. Therefore, investigating ADHD-related mechanisms in non-clinical samples can elucidate the underlying cognitive and motivational processes without the confounding effects of clinical comorbidities or medication.

Beyond attentional and inhibitory difficulties, ADHD is also associated with maladaptive decision-making styles. Decision-making impairments have been linked to a range of negative outcomes, including interpersonal difficulties and increased risk-taking behaviors, such as substance use and gambling [9–11]. To clarify how motivational and cognitive factors contribute to maladaptive decision-making, a prominent theoretical framework, the dual pathway model [12], posits two distinct but interacting routes to ADHD-related impairments. The motivational pathway, consistent with dynamic developmental theory [13], is reflected in steep delay discounting and a strong preference for immediate over delayed rewards, thought to be underpinned by dysregulated reward-processing circuits involving the ventral striatum and mesolimbic dopamine systems [14, 15]. The cognitive pathway involves deficits in executive functions, such as working memory, inhibitory control, and sustained attention, associated with altered functioning of prefrontal networks that support goal maintenance and cognitive control [15–17].

Rather than operate in isolation, motivational and cognitive deficits interact in producing maladaptive decision-making in ADHD [18–20]. Consistent with this view, recent neuroimaging and meta-analytic studies have demonstrated overlapping and interrelated activity in fronto-striatal networks during tasks involving both reward evaluation and executive control [15, 21, 22], supporting a neurobiological basis for the dual pathway model. These interacting mechanisms can be experimentally examined using neuropsychological paradigms, such as the Cambridge Gambling Task (CGT), which assesses decision-making under explicit risk [23, 24]. The CGT provides two key indices, namely, decision-making quality (DMQ) and risk adjustment (RAJ), which have been shown to load on a common factor reflecting decision-making competence [25]. Because task performance reflects both cognitive control and motivational influences, the CGT is particularly suitable for examining their combined contributions to decision-making [26, 27].

Research has demonstrated that children and adolescents with ADHD show impairments on the CGT, including reduced risk adjustment and poor decision-making quality [10, 25, 26, 28–30]. However, evidence from non-clinical adult samples is limited. No study has clarified how ADHD traits in the general population relate to CGT performance. Moreover, relatively few empirical studies have examined how motivational and cognitive processes jointly contribute to ADHD-related decision-making, despite theoretical proposals emphasizing their interaction [31]. In particular, research that integrates delay discounting, multiple executive function domains, and CGT performance within a single analytical framework is scarce. This gap is especially notable in non-clinical adult populations, where ADHD traits can be studied dimensionally rather than categorically.

The present study addresses these gaps by first modeling a general structure aimed at explaining the hypothesized relationships among motivational and cognitive tasks, and by testing a structural equation model to examine how ADHD traits are positioned within this broader structure of interrelated cognitive processes. By examining delay discounting and executive functions (working memory, sustained attention, and response inhibition) in relation to decision-making performance in the CGT, this study aims to clarify how patterns of decision-making performance are associated with elevated ADHD traits. To capture these mechanisms dimensionally within the general population, we hypothesized that steeper delay discounting and poorer executive functions would be associated with elevated ADHD traits through impaired CGT performance.

## Materials and methods

### Participants

We recruited 63 young adults (34 males, 29 females; mean age = 21.83 years; SD = 1.73 years) from local universities in Japan through convenience sampling, using posted study advertisements (posters) on campus. They received prepaid cards equivalent to JPY 1,000 as compensation. We assessed the handedness of each participant using the Japanese version of the FLANDERS Handedness Questionnaire [32]. The mean score was 8.06 (SD = 5.18), indicating a predominance of right-handed participants. Eligibility criteria were specified in the study information provided prior to participation and included: (a) age between 18 and 29 years, (b) no self-reported history of psychiatric or neurological disorders, and (c) normal or corrected-to-normal vision. Psychiatric and neurological history was assessed by self-report during the consent process; individuals who reported a history of such conditions were not enrolled in the study. No structured diagnostic interviews or medical record checks were conducted. All participants provided written informed consent prior to the experiments. This study complied with the Declaration of Helsinki and was approved by the ethics committee of Asahikawa Medical University (approval no. 23037). Data were collected between August 2023 and October 2024.

A total of 66 individuals initially participated in the study. Data from three participants were excluded from the final analysis: one duplicate participant whose second session was omitted, one participant with missing questionnaire data, and one participant who showed a non-differentiated response pattern (i.e., consistently choosing the same option in both delay discounting tasks and within a questionnaire subscale).

### Tasks and procedure

The participants completed a behavioral assessment battery consisting of two delay discounting tasks, four executive and decision-making tasks from the Cambridge Neuropsychological Test Automated Battery (CANTAB) [33]. The delay discounting tasks included the five-trial adjusting delay task (ADT-5) [34] and an adjusting-amount task based on the hyperbolic discounting model [34, 35]. We measured ADHD-related traits using the Adult ADHD Self-Report Scale (ASRS v1.1) Japanese version [36–38].

All computerized tasks were administered on a tablet PC (Apple iPad Pro, 11-inch) in a quiet experimental room. We fixed the sequence of tasks as follows: the first delay discounting task; four CANTAB tasks (Stop-Signal Task [SST], Spatial Working Memory [SWM], Rapid Visual Information Processing [RVP], and CGT); the second delay discounting task; and finally, the ASRS, which was completed on paper. We counterbalanced the order of the two delay discounting tasks across participants, such that those with even ID numbers completed the ADT-5 first and those with odd ID numbers completed the adjusting-amount task first.

#### Delay discounting tasks

We assessed intertemporal choice using the ADT-5 [34]. In each trial, the participants chose between a smaller immediate reward (JPY 50,000) and a larger delayed reward (JPY 100,000). The initial delay was set at three weeks. Based on the participant’s choice, we adjusted the delay in subsequent trials by halving the interval upwards (e.g., two years) or downwards (e.g., one day). This adjustment continued across five trials, resulting in an individualized estimate of the delay at which the subjective value of the delayed reward was 50% of its nominal value (effective delay 50%, ED50) [39]. We calculated the discounting rate (*k*) as the reciprocal of ED50 according to the hyperbolic discounting model [35]. Prior to statistical analyses, we log-transformed *k* values (log *k*) to address their tendency to be positively skewed [40].

In addition, we administered a traditional adjusting-amount delay discounting task [34, 35] for comparison. In this task, participants made hypothetical choices between an immediate reward of JPY 50,000 and a delayed reward of JPY 100,000 across six delays (one month, six months, one year, five years, 10 years, and 25 years). Subjective indifference points at each delay were determined using the adjusting-amount procedure. Delay discounting rates (*k*) were estimated by fitting a hyperbolic discounting model to these indifference points and were log-transformed prior to analysis.

Although the two tasks differed in task structure, the log-transformed *k* values derived from the ADT-5 were strongly and positively correlated with those obtained from the adjusting-amount task (*r* = 0.768, *n* = 63, *p* < 0.001; see Additional file 1: Table S1). To address potential concerns regarding nonsystematic discounting, we additionally conducted sensitivity analyses excluding participants who violated the criteria proposed by Johnson and Bickel [41]. The association between the ADT-5–derived index and the adjusting-amount–derived log *k* was significant and of similar magnitude in this quality-controlled subsample (*r* = 0.706, *n* = 48, *p* < 0.001; see Additional file 1: Table S1). On this basis, to minimize redundancy in subsequent analyses, only the ADT-5–derived delay discounting index was used in the primary models. For completeness, delay discounting indices derived from the adjusting-amount task (including both log-transformed *k* values and the area under the curve [AUC]) are reported in the Supplementary Materials (see Additional file 1: Table S1), and were further examined in supplementary regression and structural equation modeling analyses (Tables S3 and S4).

#### Stop-signal task (CANTAB-SST)

We assessed response inhibition using the SST (CANTAB-SST Basic 2.0). We asked the participants to respond as quickly and accurately as possible to a go signal (a left- or right-pointing arrow) by pressing the corresponding button on the touchscreen. In a subset of trials, a stop signal (a brief auditory beep) was presented shortly after the go signal, instructing participants to withhold their response. We used a staircase tracking algorithm to dynamically adjust the stop-signal delay and achieve approximately 50% successful inhibition. The primary outcome measure was the stop-signal reaction time (SSRT). SSRT values were derived from the CANTAB SST Basic 2.0 summary scores and estimate the covert latency of the stop process at the point where participants successfully inhibit their responses on approximately 50% of stop trials, consistent with key methodological principles outlined in current consensus guidelines on the stop-signal task [42]. The specific computational algorithm used by CANTAB to derive SSRT (mean or integration method) is not explicitly specified in the publicly available technical documentation.

#### Spatial working memory task (CANTAB-SWM)

We assessed working memory using the SWM task (CANTAB-SWM Recommended standard 2.0). In this task, participants searched for hidden tokens amid several boxes that were displayed on the screen. Once a token had been found in a box, that box would not contain another token. As such, the participants had to remember and avoid revisiting boxes that previously contained a token. The task included three set sizes (four, six, and eight boxes). The primary outcome measure was the total number of between-search errors (SWMBE), defined as returning to a box that previously contained a token. Higher SWMBE values indicated poorer working memory performance.

#### Rapid visual information processing task (CANTAB-RVP)

We assessed sustained attention using the RVP task (CANTAB-RVP 3 Targets). Participants monitored a continuous stream of digits (2–9) presented in pseudorandom order at a rate of 100 digits per minute for six minutes, during which the three predefined target sequences occurred repeatedly at pseudorandom intervals. In line with the CANTAB task specification, nine target sequences are presented per minute, yielding a total of 54 target sequences over the 6-min task. Participants were instructed to detect and respond whenever one of three predefined target sequences (e.g., 2–4–6, 3–5–7, or 4–6–8) appeared. The outcome measures were the target detection sensitivity (RVPA; A′), which reflected sustained attention, and median latency of correct responses (RVPMDL), which reflected processing speed. RVP A′ is widely used as an index of sustained visual attention in prior work employing the CANTAB RVP task [43].

#### Cambridge Gambling Task (CANTAB-CGT)

We assessed decision-making under risk using the CGT (CANTAB-CGT Ascending First Shortened). In each trial, ten boxes (colored red or blue in varying ratios) were displayed, and participants guessed under which color a hidden token was located. Participants were explicitly instructed that the ratio of colored boxes indicated the probability of the token being under each color, and that their goal was to maximize the total number of points earned across the task. After choosing a color, they placed a bet ranging from 5 to 95% of their current points. Stakes were presented in either ascending (5%, 25%, 50%, 75%, 95%) or descending order (95%, 75%, 50%, 25%, 5%). The participants pressed the screen to stop the sequence at their chosen stake.

The outcome measures were (i) decision-making quality (CGTDMQ), or the proportion of trials in which the participant bet on the more probable outcome; (ii) risk adjustment (CGTRAJ), calculated from the average proportion of points bet as a function of the number of boxes in the majority, reflecting sensitivity to risk; and (iii) delay aversion (CGTDAV), calculated as the difference in risk taking between the descending and ascending conditions (descending − ascending), with higher scores indicating a stronger tendency to bet early rather than wait.

#### Questionnaire (ASRS)

We assessed ADHD-related traits using the ASRS (v1.1), Japanese version [36–38]. The scale consists of 18 items corresponding to the DSM-IV Criterion A symptoms of inattention and hyperactivity-impulsivity. Although the ASRS v1.1 was originally developed based on DSM-IV criteria, the symptom descriptions themselves show minimal differences from those in DSM-5. Importantly, the validity of the Japanese version of the ASRS v1.1 has been confirmed against DSM-5–based ADHD diagnoses in Japanese adults [38], supporting its use as a dimensional measure of ADHD-related traits.

Part A comprises six items that were originally selected as a screening scale, whereas Part B consists of 12 additional items capturing a broader range of ADHD-related behaviors and daily-life situations. Participants rated the frequency of each symptom on a five-point Likert scale (*0* = *never* to *4* = *very often*). The maximum possible scores are 24 for Part A, 48 for Part B, and 72 for the total scale.

In the present study, we used Part A scores (ASRSA) as the primary index of ADHD traits, based on the established screening performance and predictive validity of the six-item screener in the original ASRS v1.1 [36]. The ASRS was used as a dimensional index of ADHD-related traits rather than for diagnostic classification, which is consistent with its application in non-clinical and community samples, including recent large population-based work linking ASRS trait scores to cognitive and personality characteristics [44]. In the present sample, internal consistency was modest for Part A (Cronbach’s α = 0.65), acceptable for Part B (α = 0.85), and good for the total 18-item scale (α = 0.88).

### Statistical analyses

We conducted all analyses with two-tailed tests and an α level of 0.05. Unless otherwise noted, descriptive statistics are reported as mean (*SD*). We performed the analyses using IBM SPSS Statistics 29.0 and AMOS 28.0 (IBM Corp., Armonk, NY, USA) for descriptive, correlation, regression, and structural equation modeling (SEM) analyses, and G*Power 3.1 [45] for sensitivity analyses to describe the minimal effect sizes that could be detected with adequate power given the fixed sample size.

#### Data preprocessing

Delay discounting rates (*k*) from the ADT-5 were log-transformed (log *k*) to correct for positive skew. CANTAB outcome variables (SST, SWM, RVP, CGT) were derived directly from the summary scores automatically generated by the CANTAB administration software, following the Cambridge Cognition guidelines. To simplify the SEM and capture a common dimension of decision-making performance, we combined decision-making quality (CGTDMQ) and risk adjustment (CGTRAJ) into a composite score (CGT composite) using principal component analysis (PCA). We analyzed delay aversion (CGTDAV) separately. After exclusions, all variables had complete data.

#### Correlation analyses

As an exploratory step, we examined zero-order correlations among ADHD traits (ASRSA), delay discounting (log *k*), executive-function measures, and CGT indices. Uncorrected *p*-values are reported, consistent with the exploratory nature of these analyses.

#### Multiple regression analyses

Only CGT measures showed direct zero-order associations with ASRSA. Thus, we conducted multiple regression analyses to further examine predictors of CGT performance. We specified a multiple linear regression model with the CGT composite as the dependent variable and SWMBE, RVPA, SSRT, and log *k* as simultaneous predictors. We chose these predictors based on both their theoretical relevance to executive functions and impulsive choice, as well as their observed associations in the preliminary correlation analyses. Standardized regression coefficients (*β*) are reported.

#### Structural Equation Modeling (SEM)

We specified an exploratory path model to examine the processes linking delay discounting, executive functions, decision-making, and ADHD traits. Specifically, given that log *k* (ADT-5) showed a unique correlation with sustained attention (RVPA) in the preliminary analyses, it was modeled as predicting RVPA, which in turn predicted decision-making performance (CGT composite), which then predicted ADHD traits (ASRSA). In addition, based on the significant correlation observed between RVPA and SWMBE, we included a path from RVPA to SWMBE to examine a potential sequential mediation from sustained attention to working memory errors. We also included direct paths from working memory errors (SWMBE) and response inhibition (SSRT) to the CGT composite.

All variables were treated as observed continuous indicators (path analysis). We estimated models using the maximum likelihood method in IBM AMOS 28. We evaluated indirect effects using nonparametric bootstrapping (5,000 resamples; bias-corrected percentile method). Because indirect effects represent the product of multiple paths and their sampling distribution is often non-normal, bootstrapping provides a robust test of their significance [46]. We assessed model fit using χ^2^, comparative fit index (CFI), Tucker–Lewis index (TLI), and root mean square error of approximation (RMSEA) with its 90% confidence interval and p-close. Standardized path coefficients (*β*) are reported for direct paths, and unstandardized coefficients (*B*) for bootstrapped indirect effects.

#### Sensitivity analysis

Because recruitment constraints fixed the sample size at *n* = 63, we conducted sensitivity analyses using G*Power 3.1 [45] to describe the minimal effect sizes detectable with 80% power at α = 0.05. For bivariate correlations (two-tailed, α = 0.05), the design had 80% power to detect effects of approximately |*r*|≥ 0.34. For multiple regression with four predictors, the design had 80% power to detect model-level effects of *f*^*2*^ ≥ 0.21 (*R*^*2*^ ≥ 0.17). These sensitivity estimates indicate that the study had sufficient sensitivity to detect at least moderate associations, whereas smaller effects (e.g., |*r*| ≈ 0.20) might not be reliably detected.

## Results

### Descriptive statistics

Descriptive statistics for all demographic and task performance measures are presented in Table 1. Summary statistics for ASRS Part A and Part B scores are shown in Table 1. Detailed descriptive information for ASRS scores, including total scores, is provided in the Supplementary Materials (see Additional file 1: Table S2).

**Table 1:** Participant Characteristics and Task Performance (*n* = 63)

Variables	M	SD	Description/Notes
Demographic characteristics			
Age (years)	21.83	1.73	
Sex (male/female)	34/29		
Handedness (FLANDERS score)	8.06	5.18	Higher scores = stronger right-hand preference
ADHD traits			
ASRSA	9.14	3.12	Six-item screener (used for main analyses)
ASRSB	14.75	6.83	Broad ADHD-related behaviors
Delay discounting			
ADT-5 (log *k*)	−2.78	0.80	Higher = steeper discounting
Executive function tasks (CANTAB)			
Stop-signal RT (SSRT)	307.94	36.46	Response inhibition (ms)
Spatial working memory errors (SWMBE)	4.08	6.14	Higher = poorer performance
Sustained attention (RVP task)			
RVPA	0.94	0.03	Signal detection sensitivity (A′)
RVPMDL	359.08	39.23	Median response latency (ms)
Decision-making (Cambridge Gambling Task; CGT)			
Decision-making quality (DMQ)	0.95	0.09	Proportion of rational choices
Risk adjustment (RAJ)	2.09	1.09	Sensitivity to risk
Delay aversion (DAV)	0.12	0.12	Tendency to bet early

### Correlation analyses

Table 2 presents zero-order correlations among ADHD traits (ASRSA), delay discounting (log *k*), executive functions, and CGT indices (composite performance and delay aversion). Correlations involving individual CGT subcomponents (decision-making quality and risk adjustment), ASRS Part B and total scores, as well as delay discounting indices derived from the adjusting-amount task, are reported in the Supplementary Materials (see Additional file 1: Table S1).

**Table 2:** Correlations among main study variables (*n* = 63)

Variables	1	2	3	4	5	6	7	8
1 ASRSA	—	-.012	.065	.076	-.012	-.116	-.360**	.140
2 log *k*		—	.072	.041	-.285*	-.027	-.064	.113
3 SSRT			—	-.145	-.055	-.266*	-.160	.159
4 SWMBE				—	-.279*	-.053	-.424**	-.072
5 RVPA					—	.011	.326**	-.056
6 RVPMDL						—	.226	-.142
7 CGT composite							—	-.138
8 CGT DAV								—

*p <.05, **p <.01

ASRSA was significantly correlated with CGT composite performance, but not with other cognitive measures. The CGT composite score showed significant correlations with working memory (SWMBE) and sustained attention (RVPA), which were also significantly correlated with each other. Delay discounting (log *k*) was significantly correlated with RVPA. SSRT was correlated with median latency in the RVP task (RVPMDL). Delay aversion (DAV) showed no significant correlations with ADHD traits, executive functions, or delay discounting and was therefore excluded from subsequent modeling.

CGT composite score was derived from decision-making quality and risk adjustment using PCA (see Data preprocessing section and Multiple Regression Analyses section).

### Multiple regression analyses

In the correlation analyses, CGT performance measures were associated with ASRSA. Therefore, we next examined cognitive task measures to identify which of them predicted CGT performance. This regression analysis was conducted as a preliminary step for the subsequent path model, in which cognitive tasks were expected to influence ASRSA indirectly through the CGT.

Prior to the regression, we conducted a PCA on DMQ and RAJ to derive a composite index of CGT performance and thereby simplify the subsequent model, given the modest sample size. The PCA yielded a single component (eigenvalue = 1.51), accounting for 75.4% of the variance. Both DMQ and RAJ loaded strongly on this component (loadings of 0.87 for both variables), with communalities of 0.75, indicating that the two measures reflected a common underlying dimension. This justified the creation of a CGT composite score, which was subsequently used as the dependent variable in regression analyses.

We conducted a multiple regression analysis to examine predictors of CGT composite performance. The overall model was significant, *F*(4, 58) = 5.32, *p* = 0.001, explaining 22% of the variance (adjusted *R*^*2*^ = 0.218). Among the predictors, working memory errors (SWMBE) emerged as a significant negative predictor. Sustained attention (RVPA) and response inhibition (SSRT) showed trend-level associations, whereas delay discounting (log *k*) was not a significant predictor. Table 3 presents the results of this multiple regression analysis.

**Table 3:** Multiple regression predicting CGT composite performance (*n* = 63)

Predictor	*B*	*SE B*	*β*	*p*
log *k*	0.034	0.146	0.027	.819
SSRT	–0.006	0.003	–0.208	.074
SWMBE	–0.065	0.019	–0.396	.001
RVPA	6.055	3.503	0.212	.089

Model summary: *R*^*2*^ = 0.27, Adjusted *R*^*2*^ = 0.22, *F*(4, 58) = 5.32, *p* =.001

All variance inflation factor values were < 2, indicating no multicollinearity concerns

For reference, results from an otherwise identical multiple regression model using delay discounting derived from the adjusting-amount task are reported in the Supplementary Materials (see Additional file 1: Table S3).

### Structural equation modeling

**Fig. 1:**
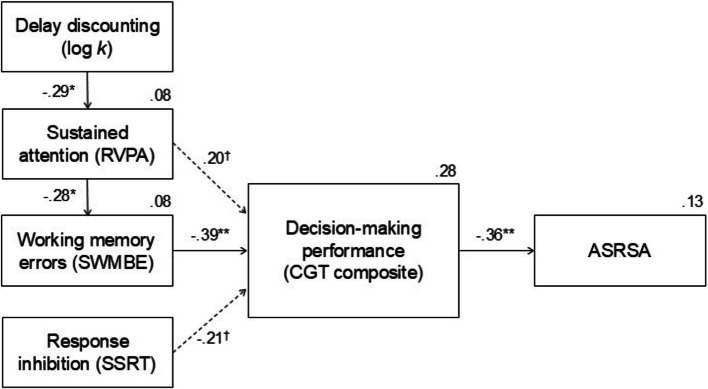
Structural equation model linking delay discounting, executive functions, decision-making, and ADHD traits. Solid lines indicate significant direct effects (*p* <.05), whereas dashed lines indicate marginal effects (.05 ≤ *p* <.10). Standardized path coefficients (*β*) are shown

Based on the preliminary findings, we tested a path model (*n* = 63) to examine hypothesized processes linking delay discounting, executive functions, decision-making, and ADHD traits. The final path model is illustrated in Fig. 1. The model fit the data well: χ^2^(9) = 3.50, *p* = 0.94, CFI = 1.00, TLI = 1.00, RMSEA = 0.00 (90% CI [0.00, 0.03], PCLOSE = 0.96).

The analysis revealed significant standardized paths from delay discounting (log *k*, ADT-5) to sustained attention (*β* = –0.285, *p* = 0.019), from sustained attention to working memory errors (*β* = −0.279, *p* = 0.022), from working memory errors to decision-making performance (*β* = −0.393, *p* < 0.001), and from decision-making performance to ADHD traits (*β* = −0.363, *p* = 0.002). In contrast, the paths from response inhibition to decision-making performance (*β* = −0.205, *p* = 0.057) and from sustained attention to decision-making performance (*β* = 0.202, *p* = 0.072) showed marginal trends toward significance.

Bootstrapped indirect effects (5,000 resamples; bias-corrected percentile method) further indicated that ADHD traits were indirectly influenced by (a) delay discounting through sustained attention and decision-making performance; (b) delay discounting through sustained attention, working memory, and decision-making performance; and (c) working memory through decision-making performance. The indirect path from response inhibition through decision-making performance to ADHD traits showed a marginal trend. The bootstrapped indirect effects are summarized in Table 4.

**Table 4:** Bootstrapped indirect effects in the structural equation model (*n* = 63)

Mediational pathway	Indirect effect (*B*)	Boot SE	95% CI	*p* value
log *k* → RVPA → CGT → ASRSA	0.081	0.074	[0.004, 0.338]	.029
log *k* → RVPA → SWMBE → CGT → ASRSA	0.044	0.043	[0.004, 0.214]	.015
SWMBE → CGT → ASRSA	0.073	0.036	[0.017, 0.162]	.006
SSRT → CGT → ASRSA	0.006	0.004	[–0.001, 0.015]	.071

For reference, standardized path coefficients from an otherwise identical structural equation model using delay discounting derived from the adjusting-amount task are reported in the Supplementary Materials (see Additional file 1: Table S4), and an additional model using the ASRS total score as the ADHD outcome is also reported there (see Additional file 1: Table S5).

## Discussion

### Summary of findings

The present study examined how ADHD traits are related to decision-making in a non-clinical population. Decision-making was assessed using the CGT, an explicit risk paradigm in which outcome probabilities are clearly specified. While this approach does not allow broad claims about decision-making processes in general, it enables a focused examination of how multiple cognitive and motivational factors jointly contribute to decision-making under explicit risk. Rather than treating CGT performance in isolation, the present study used this task as a window into the broader pattern of relationships among delay discounting, executive functions, and decision-making in a healthy population.

Interrelationships among delay discounting, sustained attention, working memory, and decision-making are expected even in non-clinical populations and should be regarded as part of a general cognitive structure rather than as ADHD-specific findings. Accordingly, the present study was designed to first capture this general pattern of interrelated cognitive processes and to then examine where ADHD traits were selectively linked within this structure, with particular attention to decision-making performance as a potential integrative pathway.

Consistent with this conceptual framework, the SEM analysis indicated that ADHD traits were indirectly associated with both delay discounting and executive functions through decision-making performance in the CGT. Specifically, weaker working memory emerged as the strongest contributor to poorer decision-making performance, reflected in reduced DMQ and RAJ scores. Importantly, working memory and sustained attention formed a sequential pathway. In this pathway, steeper delay discounting was associated with poorer sustained attention, which in turn was accompanied by greater working memory errors, poorer decision-making performance, and higher ADHD trait scores. Response inhibition showed only a marginal association with decision-making at the trend level. Taken together, these findings indicate that ADHD traits in this non-clinical sample are associated with both motivational (delay discounting) and executive (sustained attention, working memory, inhibition) pathways that converge on decision-making performance in the CGT.

### Decision-Making under explicit risk in ADHD traits

Decision-making has been implicated in a wide range of difficulties in ADHD, including risk-taking behaviors and interpersonal problems [10, 20, 26, 27, 47]. However, decision-making is a multifaceted construct, and different experimental paradigms capture distinct aspects of risky or uncertain choice. Accordingly, the present study examined decision-making using the CGT, an explicit probability-based task, to explore how ADHD traits relate to choice behavior under risk.

In the present study, both a reduction in DMQ, reflecting deviations from probabilistic choice, and lower RAJ, reflecting differences in bet adjustment as a function of explicit probabilities, were significantly correlated with ADHD trait scores in a non-clinical sample. PCA demonstrated that DMQ and RAJ strongly loaded on a single factor. We therefore combined these indices into a composite score representing overall performance on the CGT. Previous confirmatory factor analysis has similarly shown that DMQ and RAJ converge onto a common CGT-related decision-making factor [25], supporting the use of a composite index in this task-specific context. Our SEM further indicated that lower CGT composite scores were associated with higher ADHD trait scores, and that CGT performance statistically accounted for the relation between executive functions (sustained attention and working memory) and ADHD traits.

These associations are in line with previous CGT studies, in which lower DMQ and reduced RAJ have repeatedly been reported in participants diagnosed with ADHD on this task [10, 25, 26, 28, 30]. In the CGT, outcome probabilities are explicitly indicated by the ratio of colored boxes, and selecting the majority color constitutes the optimal choice. RAJ reflects how strongly bet size is modulated by these explicit probabilities and is often interpreted as an index of risk sensitivity within the CGT. However, because the optimal strategy in this task is always to bet the maximum amount, reduced RAJ does not necessarily indicate irrational decision-making per se [26]. Instead, it may capture individual differences in how participants respond to the task’s explicit rules and incentives. A longitudinal study of adolescents with ADHD showed that reduced RAJ persisted over a four-year follow-up [10], suggesting that this CGT-specific response pattern may represent a stable characteristic associated with ADHD.

The association between higher ADHD traits and lower DMQ and RAJ in the CGT may reflect several task-specific mechanisms. Rather than indicating generalized decision-making deficits, these patterns may reflect heightened sensitivity to task context or differences in how explicit probability information is processed. Pollak et al. [26] proposed that when participants are asked the “obvious question” of which option is more likely, individuals with ADHD may become suspicious about the fairness of the task, leading them to select the less probable option. In their study, this explicit probability judgment (e.g., asking “Which box do you think contains the coin?”) was removed; under these modified task conditions, no significant differences between groups were found on any CGT measure reported in their study, suggesting that task instructions may influence apparent choice patterns in explicit risk tasks.

In other decision-making paradigms that require balancing exploration and exploitation, such as multi-armed bandit tasks, ADHD has been associated with a bias toward excessive exploration [48, 49]. Within the CGT context, choosing the minority option may similarly reflect exploratory responding in a context where probabilities are explicitly known. This interpretation remains tentative, but is consistent with the trend-level association observed in the present study between longer SSRTs, reflecting weaker response inhibition, and lower CGT composite scores. Future research using multiple decision-making paradigms, including bandit tasks and reinforcement learning models, will be necessary to rigorously test this account. This will clarify whether the hypothesized exploratory choices observed in the CGT reflect a broader exploration bias in ADHD or are specific to tasks with explicit probability structures.

### Role of executive functions: focus on working memory

Working memory also played an important role within the pathway linking executive functions, decision-making performance, and ADHD traits. Weaknesses in working memory and decision-making (e.g., risk seeking and impulsivity) are often observed simultaneously in individuals with ADHD. In tasks such as the CGT, participants are required to retain information about previous choices and their outcomes while making subsequent decisions; as such, working memory substantially contributes to task performance. Indeed, adults with ADHD show poorer performance in deliberative decision-making tasks that require working memory (i.e., Adult Decision-Making Competence, which involves solving problems by applying predetermined decision rules, such as selecting consumer products), and logistic regression analysis has indicated that this deficit is the strongest discriminator between ADHD and control groups [50]. Therefore, working memory appears to be an important contributor to decision-making difficulties observed in adults with ADHD. Even in typically developing adults, imposing a high working memory load has been shown to impair performance on the Iowa Gambling Task and to attenuate the implicit learning effect, which refers to the gradual tendency to learn advantageous decks from the history of card selections [51].

A previous study confirmed that working memory training with the n-back task improves early attentional processes in the ADHD group and reduces their tendency to favor high-stakes betting, an index of risk-seeking behavior in a gambling task analogous to the CGT [52]. Furthermore, our SEM results indicated that weaker working memory was indirectly associated with higher ADHD trait scores through poorer CGT performance (i.e., reduced DMQ and RAJ scores), a pattern consistent with previous findings. Importantly, working memory performance was also associated with sustained attention, forming part of a sequential pathway linking sustained attention, decision-making performance, and ADHD trait scores.

Taken together, these results suggest that working memory plays a key role within the network of executive and decision-making processes associated with individual differences in ADHD traits. These findings are consistent with theoretical accounts that emphasize the role of executive functioning within the dual-pathway model of ADHD proposed by Sonuga-Barke [12].

### Indirect pathways linking delay discounting, sustained attention, decision-making, and ADHD traits

Our study identified a significant negative correlation between delay discounting and sustained attention. Delay discounting was significantly correlated only with sustained attention, and on this basis, delay discounting was modeled as preceding sustained attention within the SEM framework. The analysis showed a significant indirect association linking steeper discounting to higher ADHD trait scores through reduced sustained attention and poorer decision-making performance. Previous studies on ADHD have repeatedly reported that both reduced sustained attention and steep delay discounting are commonly observed characteristics [53]. Similarly, in the field of substance dependence, both delay discounting and performance in sustained attention/inhibitory control tasks, such as the continuous performance task (CPT), have been shown to be associated with treatment outcomes [54]. However, relatively few studies have statistically examined the direct relation between delay discounting and sustained attention. Neuroimaging studies have reported overlapping activation in the anterior prefrontal cortex during both working memory and delay discounting tasks [55–57]. Given that working memory tasks inherently require sustained attention, these results can also be interpreted from an attentional control perspective. Consistent with this view, our data showed a significant negative correlation between working memory error (SWMBE) and sustained attention (RVPA). Our SEM further indicated a close association between sustained attention and working memory performance. Taken together, these findings suggest that steeper delay discounting is consistently related to individual differences in sustained attention and working memory.

Both sustained attention tasks (e.g., CPT and RVP), which require individuals to “wait” for target stimuli, and delay discounting tasks, which require individuals to “wait” for delayed rewards, share a common temporal demand. Difficulties in sustained attention (e.g., premature or missed responses) and steep discounting may therefore reflect overlapping tendencies related to waiting, consistent with the concept of delay aversion in ADHD. In this context, the pattern observed in our SEM, in which steeper delay discounting was associated with reduced sustained attention, is consistent with Sagvolden et al.’s hypothesis that a steep delay-of-reinforcement gradient may contribute to attentional difficulties [13].

In addition, the association between sustained attention and decision-making performance observed in our SEM is consistent with a meta-analysis of adult ADHD studies examining the relation between attentional function and decision-making characteristics [19]. This meta-analysis reported that poorer decision-making performance (i.e., a tendency to fail to select advantageous options in the Iowa Gambling Task) and reduced sustained attention (i.e., commission errors, omission errors, and increased reaction time variability in the CPT) tend to co-occur in adults with ADHD with effect sizes of similar magnitude. However, the same study did not address the directional relation between the two domains. From a theoretical perspective, decision-making tasks such as the CGT rely on attentional resources, including the maintenance of probabilistic information and response control, as well as on working memory. Consistent with this view, our findings suggest that individual differences in sustained attention are associated with decision-making performance, both directly and indirectly through working memory efficiency. Accordingly, sustained attention may be considered as an important component within the network of processes linked to decision-making performance. Our SEM results further indicate that reduced sustained attention is indirectly associated with higher ADHD trait scores via poorer decision-making quality.

Neuroimaging evidence from event-related fMRI studies of the CGT indicates that decision phases engage regions implicated in attentional control, such as the dorsolateral prefrontal cortex, anterior cingulate cortex, and thalamus, in addition to reward-related structures [58]. These findings suggest that CGT performance relies not only on valuation and reward-related processes but also on sustained attention and executive monitoring, consistent with the view that attentional processes are closely integrated with decision-making performance.

### Additional considerations: delay aversion

Increased delay aversion (DAV) on the CGT has often been reported as a frequently observed characteristic in ADHD [25]. However, in our study, DAV was not significantly associated with ADHD traits, other CGT indices, or executive function measures. In a large longitudinal study of a general youth sample (ages 11–14 years), DAV was found to be reciprocally related to inattentive/hyperactive tendencies and conduct problems [59]. Sørensen et al. [10] further showed that higher baseline DAV was associated with interpersonal problems in children with ADHD. Notably, unlike risk adjustment (RAJ), this group difference disappeared at the four-year follow-up (mean age = 14.5 years). Taken together, these findings suggest that DAV on the CGT may characterize a subset of individuals with ADHD but may not represent a developmentally stable trait. The absence of an association between DAV and ADHD traits in our non-clinical university sample is consistent with this interpretation.

The lack of associations between executive function measures and DAV is also in line with previous findings. Using confirmatory factor analysis in medication-naïve children with ADHD, Coghill et al. [25] identified DAV as an independent factor, distinct from executive functions, such as working memory, inhibition, and timing. Likewise, Flouri et al. [60] reported that general cognitive resources (IQ) predict RAJ and DMQ, but not DAV, further supporting the view that DAV reflects a motivational construct separable from cognitive control processes.

In our study, DAV was not correlated with delay discounting. Whereas delay discounting estimates a relatively stable parameter of temporal preference across multiple choices, DAV captures context-dependent affective responses to waiting in a gambling situation. Accordingly, DAV and log *k* may not be expected to show strong associations. These considerations suggest that in healthy adult populations, DAV may show weak or inconsistent associations with other measures. DAV on the CGT may therefore be more appropriately conceptualized not as part of a unified decision-making factor but as an independent motivational index, which may be particularly relevant in clinical ADHD, where affective components of delay intolerance are more pronounced.

### Theoretical and practical implications

We found that ADHD traits were associated with differences in decision-making performance among typically developing adults. Using SEM, we further observed that this association was statistically mediated by executive function measures, including working memory, sustained attention, and response inhibition. We also found that steeper delay discounting was related to poorer sustained attention.

Although our SEM is based on cross-sectional associations, its pattern is consistent with the idea that interventions targeting executive functions may improve decision-making performance, which could in turn be relevant to difficulties commonly associated with ADHD. Training programs that strengthen working memory and attention have been shown to reduce delay discounting [61] and suppress risk-seeking behavior, supporting the value of non-pharmacological approaches. Pharmacological treatments can also enhance decision-making: stimulants such as methylphenidate improve sustained attention [62] and attenuate delay discounting [63]. Thus, combining medication with cognitive training may represent a promising approach for addressing risk-taking and social difficulties in clinical ADHD. The observed pattern in our SEM suggests that targeting delay discounting, for instance, through episodic future thinking [64], could indirectly influence sustained attention and decision-making performance. Accordingly, motivational interventions targeting temporal preference may have the potential to support executive control and decision-making processes.

### Limitations

This study has a few limitations. First, the sample consisted of a convenience group of university and graduate students in Japan, which may limit the generalizability of the findings to broader age ranges or clinical populations. In addition, psychiatric/neurological history was assessed by self‑report, and no formal diagnostic interviews were conducted; this should be considered when interpreting the findings. Second, although the sample size (*n* = 63) was sufficient to detect medium-to-large effects at the model level, sensitivity analyses indicated limited power for small effects. Therefore, nonsignificant or trend-level associations should be interpreted with caution. Third, because of this limitation, we interpreted the path model results cautiously, considering both significance levels and bootstrap confidence intervals. Replication in clinical and other diverse populations, and longitudinal examination of potential causal relations, will be important in future work.

In addition, ADHD traits were primarily indexed using ASRS Part A in the main analyses. Although ASRS Part A is widely used as a sensitive screening measure, ASRS Part B also captures ADHD-related behaviors and could, in principle, have been included in the main outcome. To address this issue, supplementary analyses using the ASRS total score were conducted, confirming that the overall pattern of results was generally comparable to that of the primary analyses (see Additional file 1: Table S5). As a limitation, when delay discounting was indexed using the adjusting-amount–derived log *k* (see Additional file 1: Table S4), the association between delay discounting and sustained attention did not reach statistical significance, which may reflect task- or measurement-specific differences in sensitivity across delay discounting measures. Further investigation is needed to clarify the extent to which such task-specific factors influence this association. Furthermore, extending beyond the CGT to include other decision-making paradigms, such as the Iowa Gambling Task or bandit tasks, will be necessary to determine the extent to which the observed links between executive functions and decision-making generalize across tasks.

## Conclusion

The present study is consistent with a multi-pathway account of ADHD traits and points to decision-making under explicit risk as an important mediating process within this framework. Although CGT-derived delay aversion did not play a significant role, delay discounting and executive functions contributed indirectly to ADHD traits through their associations with decision-making performance. By integrating motivational and executive processes within a single structural model, the present findings advance current understanding of ADHD-related decision processes and provide a basis for future assessment-focused research.

## Supplementary Information

Supplementary Material 1: Table S1. Correlation matrix of study variables, including delay discounting indices derived from the adjusting-amount task (*n*= 63). Table S2. Descriptive statistics of Adult ADHD Self-Report Scale (ASRS) scores (*n* = 63). Table S3. Multiple regression analysis of CGT composite performance using a delay discounting index derived from the adjusting-amount task (*n* = 63). Table S4. Standardized path coefficients for the alternative SEM using delay discounting derived from the adjusting-amount task (*n* = 63). Table S5. Standardized path coefficients for the alternative SEM using ASRS total score (*n* = 63). 

## Data Availability

The datasets generated and analyzed during the current study are available from the corresponding author on reasonable request.

## Abbreviations

ADHD: Attention-deficit/hyperactivity disorder
ASRS: Adult ADHD Self-Report Scale
CGT: Cambridge Gambling Task
DMQ: Decision-Making Quality
RAJ: Risk Adjustment
DAV: Delay Aversion
ADT-5: 5-Trial Adjusting Delay Task
AUC: Area Under the Curve
SWM: Spatial Working Memory
RVP: Rapid Visual Information Processing
SST: Stop Signal Task
SEM: Structural Equation Modeling
