# Supplementary material for: Executive and motivational pathways to ADHD traits in the general population: a structural equation model of working memory, attention, delay discounting, and decision-making
Source: BMC Psychol. 2026 Mar 6;14:525. doi: 10.1186/s40359-026-04279-x (PMC13077841; doi:10.1186/s40359-026-04279-x)
Supplement: Supplementary file 1 — Supplementary Material 1: Table S1. Correlation matrix of study variables, including delay discounting indices derived from the adjusting-amount task (n= 63). Table S2. Descriptive statistics of Adult ADHD Self-Report Scale (ASRS) scores (n = 63). Table S3. Multiple regression analysis of CGT composite performance using a delay discounting index derived from the adjusting-amount task (n = 63). Table S4. Standardized path coefficients for the alternative SEM using delay discounting derived from the adjusting-amount task (n = 63). Table S5. Standardized path coefficients for the alternative SEM using ASRS total score (n = 63). [file 40359_2026_4279_MOESM1_ESM.docx]

**Supplementary Tables**

The Supplementary Tables present additional descriptive statistics and supplementary analyses to support interpretation of the main analyses. They include a full correlation matrix of all study variables (Table S1), detailed descriptive statistics for ASRS scores (Table S2), multiple regression analyses using delay discounting derived from the adjusting-amount task (Table S3), an alternative structural equation model based on this delay discounting index (Table S4), and an alternative structural equation model based on ASRS total score (Table S5).

**Table S1. Correlation matrix of study variables, including delay discounting indices derived from the adjusting-amount task (*n* = 63)**

| Variables | 1 | 2 | 3 | 4 | 5 | 6 | 7 | 8 | 9 | 10 | 11 | 12 | 13 | 14 |
| --- | --- | --- | --- | --- | --- | --- | --- | --- | --- | --- | --- | --- | --- | --- |
| 1. ASRSA | — | .749** | .877** | -.120 | .192 | -.012 | .065 | .076 | -.012 | -.116 | -.328** | -.297* | .140 | -.360** |
| 2. ASRSB |  | — | .976** | -.221 | .227 | -.197 | .051 | .056 | -.022 | -.239 | -.225 | -.150 | .019 | -.216 |
| 3. ASRS total |  |  | — | -.200 | .229 | -.148 | .058 | .066 | -.020 | -.213 | -.273* | -.208 | .061 | -.277* |
| 4. log *k* (adjusting-amount) |  |  |  | — | -.915** | .768** | -.070 | .024 | -.197 | .046 | -.064 | .021 | .101 | -.025 |
| 5. AUC (adjusting-amount) |  |  |  |  | — | -.708** | .103 | .006 | .178 | -.063 | .005 | -.002 | -.102 | .001 |
| 6. log *k* (ADT-5) |  |  |  |  |  | — | .072 | .041 | -.285* | -.027 | -.050 | -.062 | .113 | -.064 |
| 7. SSRT |  |  |  |  |  |  | — | -.145 | -.055 | -.266* | -.127 | -.151 | .159 | -.160 |
| 8. SWMBE |  |  |  |  |  |  |  | — | -.279* | -.053 | -.307* | -.429** | -.072 | -.424** |
| 9. RVPA |  |  |  |  |  |  |  |  | — | .011 | .311* | .254* | -.056 | .326** |
| 10. RVPMDL |  |  |  |  |  |  |  |  |  | — | .213 | .179 | -.142 | .226 |
| 11. CGT DMQ |  |  |  |  |  |  |  |  |  |  | — | .507** | -.145 | .868** |
| 12. CGT RAJ |  |  |  |  |  |  |  |  |  |  |  | — | -.094 | .868** |
| 13. CGT DAV |  |  |  |  |  |  |  |  |  |  |  |  | — | -.138 |
| 14. CGT composite |  |  |  |  |  |  |  |  |  |  |  |  |  | — |

Note.

Log-transformed *k* values were estimated using a hyperbolic discounting model for both the ADT-5 and the adjusting-amount task. The area under the curve (AUC) was calculated from indifference points obtained in the adjusting-amount task. Higher log *k* values and lower AUC values indicate steeper delay discounting. Descriptive statistics for the adjusting-amount–derived indices were as follows: log *k*, M = −3.23, SD = 1.26; AUC, M = 0.40, SD = 0.33. To address potential nonsystematic discounting, sensitivity analyses were conducted excluding participants who violated the criteria proposed by Johnson and Bickel [41]. The correlation between ADT-5–derived log *k* and adjusting-amount–derived log *k* remained significant and of similar magnitude in this quality-controlled subsample (*n* = 48, *r* = .706, *p* < .001).

**p* < .05, ***p* < .01.

**Table S2. Descriptive statistics of Adult ADHD Self-Report Scale (ASRS) scores (*n* = 63)**

This table reports descriptive statistics for the Adult ADHD Self-Report Scale (ASRS) Part A, Part B, and total scores in a non-clinical university student sample. Mean scores, standard deviations, observed ranges, and possible score ranges are presented for each index. Internal consistency coefficients for each scale are reported in the main text.

| **Variable** | **Mean** | **SD** | **Min–Max** | **Possible range** |
| --- | --- | --- | --- | --- |
| ASRS Part A | 9.14 | 3.12 | 4–16 | 0–24 |
| ASRS Part B | 14.75 | 6.83 | 3–35 | 0–48 |
| ASRS Total | 23.89 | 9.40 | 8–49 | 0–72 |

Note.

ASRS = Adult ADHD Self-Report Scale (v1.1).

Part A consists of six items commonly used as a screening measure, whereas Part B includes additional items assessing broader ADHD-related behaviors.

Total scores represent the sum of all 18 items.

All items were rated on a 5-point Likert scale ranging from 0 to 4.

**Table S3. Multiple regression analysis of CGT composite performance using a delay discounting index derived from the adjusting-amount task (*n* = 63)**

This table reports the results of a multiple regression analysis predicting CGT composite performance using a delay discounting index derived from the adjusting-amount task (log-transformed *k*), together with executive function and sustained attention measures. The model specification was identical to that of the primary regression analysis reported in the main text, except that the ADT-5–derived delay discounting index was replaced by the adjusting-amount–derived index.

| **Predictor** | ***B*** | ***SE*** | ***β*** | ***t*** | ***p*** |
| --- | --- | --- | --- | --- | --- |
| log *k* (adjusting-amount) | 0.009 | 0.092 | 0.011 | 0.095 | .925 |
| SSRT | −0.006 | 0.003 | −0.206 | −1.80 | .078 |
| SWMBE | −0.065 | 0.019 | −0.396 | −3.34 | .001 |
| RVPA | 5.898 | 3.439 | 0.206 | 1.72 | .092 |

Model statistics:

*R* = .518, *R²* = .268, Adjusted *R²* = .217, *F*(4, 58) = 5.31, *p* = .001

Note.

CGT = Cambridge Gambling Task; SWMBE = spatial working memory between-search errors; SSRT = stop-signal reaction time; RVPA = sustained attention (RVP A′).

Delay discounting was indexed using log-transformed *k* values derived from the adjusting-amount task.

**Table S4. Standardized path coefficients for the alternative SEM using delay discounting derived from the adjusting-amount task (*n* = 63)**

This table reports standardized path coefficients (*β*) and *p*-values for an alternative structural equation model in which the delay discounting index derived from the adjusting-amount task (log-transformed *k*) was used in place of the ADT-5–derived index. The model structure was identical to that of the primary SEM reported in the main text.

| **Path** | **Standardized *β*** | ***p*** |
| --- | --- | --- |
| log *k* (adjusting-amount)→ RVPA | −0.197 | .114 |
| RVPA → SWMBE | −0.279 | .022 |
| SSTSSRT → CGT_composite | −0.205 | .057 |
| SWMBE → CGT_composite | −0.393 | < .001 |
| RVPA → CGT_composite | 0.202 | .072 |
| CGT_composite → ASRSA | −0.363 | .002 |

Note.

Model fit indices for the alternative SEM were χ²(9) = 4.64, *p* = .86, CFI = 1.00 and RMSEA = 0.00.

**Table S5. Standardized path coefficients for the alternative SEM using ASRS total score (*n* = 63)**

This table reports standardized path coefficients (*β*) and *p*-values for an alternative structural equation model in which the total score of the Adult ADHD Self-Report Scale (ASRS total) was used as the outcome variable in place of the ASRS screener score (ASRSA). The model structure was identical to that of the primary SEM reported in the main text, with delay discounting indexed by the log-transformed *k* derived from the ADT-5.

| **Path** | **Standardized *β*** | **p** |
| --- | --- | --- |
| log *k* (ADT-5) → RVPA | −0.285 | .019 |
| RVPA → SWMBE | −0.279 | .022 |
| SSTSSRT → CGT_composite | −0.205 | .057 |
| SWMBE → CGT_composite | −0.393 | < .001 |
| RVPA → CGT_composite | 0.202 | .072 |
| CGT_composite → ASRS total | −0.279 | .022 |

Note.

Model fit indices for the alternative SEM were χ²(9) = 4.39, *p* = .88, CFI = 1.00 and RMSEA = 0.00.
